# Transducin β-Subunit Can Interact with Multiple G-Protein γ-Subunits to Enable Light Detection by Rod Photoreceptors

**DOI:** 10.1523/ENEURO.0144-18.2018

**Published:** 2018-06-11

**Authors:** Paige M. Dexter, Ekaterina S. Lobanova, Stella Finkelstein, William J. Spencer, Nikolai P. Skiba, Vadim Y. Arshavsky

**Affiliations:** 1 Department of Pharmacology and Cancer Biology, Duke University School of Medicine, Durham, North Carolina 27710; 2 Albert Eye Research Institute, Duke University, Durham, North Carolina 27710

**Keywords:** G-protein, phototransduction, retinal degeneration, transducin

## Abstract

The heterotrimeric G-protein transducin mediates visual signaling in vertebrate photoreceptor cells. Many aspects of the function of transducin were learned from knock-out mice lacking its individual subunits. Of particular interest is the knockout of its rod-specific γ-subunit (Gγ_1_). Two studies using independently generated mice documented that this knockout results in a considerable >60-fold reduction in the light sensitivity of affected rods, but provided different interpretations of how the remaining α-subunit (Gα_t_) mediates phototransduction without its cognate Gβ_1_γ_1_-subunit partner. One study found that the light sensitivity reduction matched a corresponding reduction in Gα_t_ content in the light-sensing rod outer segments and proposed that Gα_t_ activation is supported by remaining Gβ_1_ associating with other Gγ subunits naturally expressed in photoreceptors. In contrast, the second study reported the same light sensitivity loss but a much lower, only approximately sixfold, reduction of Gα_t_ and proposed that the light responses of these rods do not require Gβγ at all. To resolve this controversy and elucidate the mechanism driving visual signaling in Gγ_1_ knock-out rods, we analyzed both mouse lines side by side. We first determined that the outer segments of both mice have identical Gα_t_ content, which is reduced ∼65-fold from the wild-type (WT) level. We further demonstrated that the remaining Gβ_1_ is present in a complex with endogenous Gγ_2_ and Gγ_3_ subunits and that these complexes exist in wild-type rods as well. Together, these results argue against the idea that Gα_t_ alone supports light responses of Gγ_1_ knock-out rods and suggest that Gβ_1_γ_1_ is not unique in its ability to mediate vertebrate phototransduction.

## Visual Overview

**Figure:**
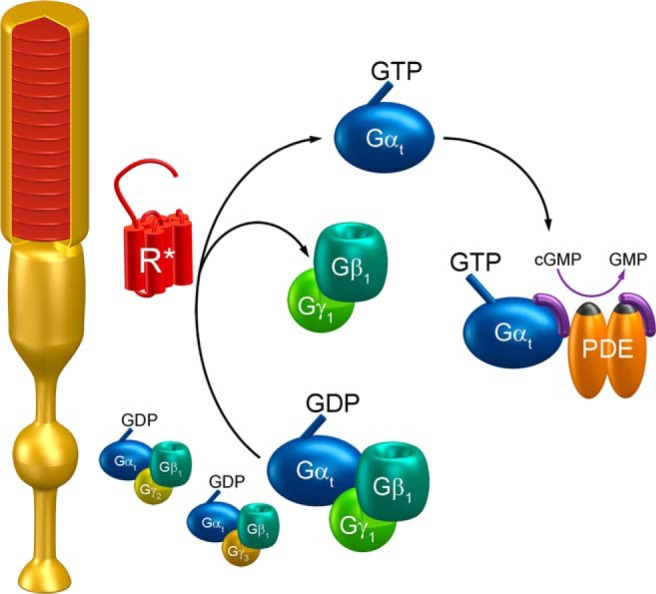

## Significance Statement

Phototransduction has been a valuable system for understanding the basic principles of G-protein signaling. One question that has remained unanswered is whether the G-protein α-subunit can support signaling without its cognate βγ partner complex. Previous studies investigating this question in photoreceptors of Gγ_1_ knock-out mice came to mutually exclusive conclusions. We now resolve this controversy by showing that phototransduction in this knockout is supported by alternative βγ complexes rather than the α-subunit alone. Most importantly, this study highlights the functional interchangeability of different γ-subunits in the context of an intact *in vivo* system.

## Introduction

The heterotrimeric G-protein transducin mediates visual signal transduction in the outer segments of vertebrate photoreceptor cells. The visual signal, or photoresponse, is initiated when photoexcited rhodopsin activates the transducin heterotrimer by catalyzing GDP−GTP exchange on its α-subunit (Gα_t_). Gα_t_ subsequently dissociates from the βγ-subunit complex (consisting of Gβ_1_ and Gγ_1_) to stimulate the downstream effector type 6 cGMP phosphodiesterase. The resulting reduction in cytosolic cGMP causes cGMP-gated ion channel closure, photoreceptor hyperpolarization, and decreased glutamate release from the synaptic terminal (for review, see Arshavsky and Burns, 2012). This signal is processed further by downstream retinal neurons before being transmitted to the brain.

Previous studies have demonstrated that knocking out individual transducin subunits produces quite different phenotypes. For example, the rods of Gα_t_ knock-out mice are completely insensitive to light (Calvert et al., 2000), while Gγ_1_ knock-out rods retain distinct light sensitivity despite the absence of this critical component of the transducin heterotrimer (Lobanova et al., 2008; Kolesnikov et al., 2011). Next, the absence of Gα_t_ does not affect the expression levels of its cognate Gβ_1_γ_1_ complex (Calvert et al., 2000; Lobanova et al., 2008), whereas the expression levels of both Gα_t_ and Gβ_1_ are drastically reduced in the Gγ_1_ knockout (Lobanova et al., 2008; Kolesnikov et al., 2011). Finally, the knockout of Gα_t_ does not affect photoreceptor viability (Calvert et al., 2000), whereas Gγ_1_ knock-out mice undergo progressive photoreceptor degeneration (Lobanova et al., 2008; Kolesnikov et al., 2011). The latter originates from proteostatic stress arising from the requirement to degrade vast amounts of Gβ_1_, unable to fold without its Gγ_1_ partner (Lobanova et al., 2013).

In a previous attempt to understand the nature of light responses in *Gγ_1_^−/−^* rods, the reduction in transducin subunits in their outer segments was correlated with the reduction in their light sensitivity (Lobanova et al., 2008). *Gγ_1_^−/−^* outer segments were shown to contain ∼50-fold less Gα_t_ and Gβ_1_ than WT rods, a reduction very close to the ∼67-fold loss in their light sensitivity also reported in that study. Because photoreceptor light sensitivity is directly proportional to the rate of transducin activation, which in turn is proportional to its concentration on outer segment discs (Pugh and Lamb, 1993; Heck and Hofmann, 2001; Sokolov et al., 2002; Arshavsky and Burns, 2014), it was suggested that *Gγ_1_^−/−^* light responses are conveyed by transducin heterotrimer using an alternate G-protein γ-subunit to enable its efficient activation by rhodopsin. However, attempts to identify this replacement Gγ were unsuccessful due to the low transducin content in these retinas.

More recently, an alternative *Gγ_1_^−/−^* mouse with a transgenic design different from that used by Lobanova et al. (2008) was characterized by Kolesnikov et al. (2011). Notably, rod photoresponses recorded from these mice had characteristics very similar to those described by the first study, including a 90-fold reduction in light sensitivity from the WT level. However, the outer segment content of Gα_t_ was estimated to be only sixfold lower than normal. Based on previously published evidence that rhodopsin can activate Gα_t_ *in vitro* without Gβ_1_γ_1_, although significantly slower than normally (Fung, 1983; Kisselev et al., 1999; Herrmann et al., 2006), the authors argued that *Gγ_1_^−/−^* rod photoresponses are conveyed by Gα_t_ acting alone. They also described a slower progression of photoreceptor degeneration in *Gγ_1_^−/−^* retinas than observed by Lobanova et al. (2008), which was attributed to differences in the genetic backgrounds of these strains.

To settle these discrepancies and elucidate the mechanism driving visual signaling in *Gγ_1_^−/−^* rods, we performed a side-by-side analysis of both *Gγ_1_^−/−^* mouse strains. First, we compared the amounts of Gα_t_ and Gβ_1_ in retinal lysates of these strains and found that the decrease in expression levels of both subunits was identical and corresponded to the decrease initially reported by Lobanova et al. (2008). Most importantly, this decrease was comparable to the decrease in rod photoresponse sensitivity reported for both strains. We next demonstrated that the remaining Gβ_1_ is present in a complex with endogenous Gγ_2_ and Gγ_3_ subunits. These results indicate that Gβ_1_γ_1_ is not the sole Gβγ complex able to facilitate phototransduction and that Gβ_1_ associated with alternative, noncanonical Gγ subunits supports transducin activation in *Gγ_1_^−/−^* rods at an efficiency comparable to that of the canonical Gβ_1_γ_1_ complex.

Given the significant difference reported in the progression of photoreceptor degeneration for the two *Gγ_1_^−/−^* strains, we also performed a systematic analysis of retinal degeneration in these animals. We have found that photoreceptors of these mice degenerate at nearly the same rate, and both strains display phenotypes consistent with abnormal proteostasis in their rods.

## Materials and Methods

### Animals

Mouse care and experiments were performed in accordance with procedures approved by the Institutional Animal Care and Use Committee of Duke University. The Deltagen Gγ_1_ knockout was licensed from Deltagen Inc. (San Mateo, CA) (Target ID 408) and was previously characterized in the study by Lobanova et al. (2008, 2013). In this mouse, regions of the Gγ_1_ coding sequence (amino acids 17–44 and intron 2) were replaced with a 6.9 kb IRES-lacZ reporter and neomycin resistance cassette. The StL Gγ_1_ knockout, previously characterized in the study by Kolesnikov et al. (2011), was provided by Dr. O.G. Kisselev (Saint Louis University, St. Louis, MO). In this mouse, the targeting construct replaced all three exons with a Neo cassette to eliminate the coding region of Gγ_1_. Transgenic mice heterozygously expressing the Ub^G76V^-GFP reporter are described in the study by Lindsten et al. (2003). WT mice used in this study were C57BL/6J from The Jackson Laboratory. None of the mouse lines contained the Rd8 mutation. Mice of either sex were used for all experiments.

### Antibodies

Rabbit anti-Gα_t_ (sc-389), anti-Gβ_1_ (sc-379), anti-Gγ_2_ (sc-374), and anti-Gγ_3_ (sc-375) antibodies, and mouse anti-Gγ_2_ (sc-134344) and anti-β-actin (sc-47778) antibodies were from Santa Cruz Biotechnology. Rabbit anti-PSMD1 (ab140682) antibody was from Abcam. Rabbit anti-Gβ_1_ (GTX114442) was from GeneTex. The specificity of the anti-Gα_t_ antibody in the context of retinal tissue was directly tested in Gα_t_ knock-out animals. The specificity of other antibodies was assumed per manufacturer descriptions. Secondary goat or donkey antibodies for Western blotting conjugated with Alexa Fluor 680 and 800 were from Invitrogen. Protein bands were visualized and quantified using the Odyssey Infrared Imaging System (LI-COR Biosciences).

### Western blotting

For quantitative Western blot analysis of Gα_t_ and Gβ_1_ protein levels, two mouse retinas per sample were solubilized in 150 μl of 1% Triton X-100 in PBS. Total protein concentration was measured using the DC Protein Assay kit (Bio-Rad), and samples were diluted with SDS-PAGE sample buffer to achieve a protein concentration of 1 μg/μl. Aliquots from Gγ_1_ knock-out mice containing 10 μg of total protein were separated by SDS-PAGE along with a serial dilution of WT retinal lysate.

### Histology and microscopy

Agarose-embedded retinal cross sections were prepared as previously described (Lobanova et al., 2010), collected in 24-well plates, and incubated for 2 h with Alexa Fluor 594 conjugate of wheat germ agglutinin (Invitrogen) in PBS containing 0.1% Triton X-100. Sections were washed three times in PBS, mounted with Fluoromount G (Electron Microscopy Sciences) under glass coverslips, and visualized using a Nikon Eclipse 90i Confocal Microscope.

Plastic-embedded retinal cross sections (1 μm thick) were prepared as previously described (Sokolov et al., 2004) and stained with toluidine blue for light microscopy. Tiled images of whole retina cross sections were obtained using the Olympus IX-81 Inverted Fluorescence Microscope, and aligned and stitched using the Olympus cellSens Dimension software. The number of photoreceptor nuclei in representative segments of outer nuclear layer (ONL) was quantified as a quantitative measure of surviving photoreceptors. The number of nuclei in a 400 μm segment of the ONL, located at 1 mm from each side of the optic nerve, was counted by hand.

### Rod outer segment isolation

Rod outer segments were isolated from WT and *Gγ_1_^−/−^* retinas as previously described (Tsang et al., 1998), with minor modifications. Briefly, retinas from 6–10 animals were removed from the eyecups and placed in 150 μl of 8% OptiPrep Density Gradient Medium (Sigma-Aldrich) in mouse Ringer’s buffer (130 mm NaCl, 3.6 mm KCl, 2.4 mm MgCl_2_, 1.2 mm CaCl_2_, 10 mm HEPES, and 0.02 mm EDTA, pH 7.4). The tubes were vortexed at maximum speed for 60 s and centrifuged at 200 × *g* for 60 s, and the supernatant containing rod outer segments was gently collected. Two hundred microliters of fresh 8% OptiPrep solution was added to the retinal pellet, and vortexing/sedimentation was performed again. This sequence was repeated at least five times. The combined supernatant was loaded on a step gradient made with 10% and 18% OptiPrep in a 4 ml centrifuge tube and centrifuged in a swing-bucket rotor at 115,000 × *g* for 30 min. Rod outer segments were collected from the interface between 10% and 18% OptiPrep, diluted with 4 ml of Ringer’s solution, and centrifuged at 100,000 × *g* for 1 h. The pellet containing rod outer segments was rinsed once with 200 μl of Ringer’s solution, resuspended in 200 μl of PBS, snap frozen in liquid N_2_, and stored at −80°C until use.

### Immunoprecipitation

Samples were prepared for immunoprecipitation as previously described (Pearring et al., 2015). Briefly, purified rod outer segments were thawed and their protein content was determined using the DC Protein Assay Kit. Samples were diluted to 0.5 μg/μl protein in the immunoprecipitation buffer (0.1% *n*-dodecyl-β-maltoside in PBS with protease inhibitor cocktail, Sigma-Aldrich), vortexed, and centrifuged at 108,000 × *g* for 30 min. The supernatant was removed for use in immunoprecipitation. Protein A Mag Sepharose beads (catalog #28944006, GE Healthcare Life Sciences) and Protein G Mag Sepharose Xtra beads (catalog #28967066, GE Healthcare Life Sciences) were used for immunoprecipitation with rabbit and mouse antibodies, respectively. In both cases, 5 μl beads were incubated with 5 μg of antibody for 2 h under rotation at room temperature. The beads were rinsed and incubated with 12.5 μg of rod outer segment lysate overnight under rotation at 4°C. After the beads were rinsed, bound proteins were eluted by boiling in the SDS-PAGE loading buffer (2% SDS) at 95°C for 10 min and analyzed by Western blotting.

### Mass spectrometry

Peptide mixes obtained from in-gel tryptic digests were analyzed using a nanoACQUITY UPLC System coupled to a Synapt G2 Mass Spectrometer (Waters). Peptides were separated on a 75 μm × 150 mm column with 1.7 μm C18 BEH (Ethylene Bridged Hybrid) particles (Waters) using a 90 min gradient of 6–32% acetonitrile with 0.1% formic acid at a flow rate of 0.3 μl/min at 35°C. For each sample, we conducted data-dependent analysis (DDA) using a 0.8 s mass spectrometry (MS) scan followed by tandem MS (MS/MS) acquisition on the top three ions. MS/MS scans for each ion used an isolation window of ∼3 Da and a dynamic exclusion window of 90 s within 1.2 Da. DDA data were converted to searchable files using ProteinLynx Global Server 2.5.1 (Waters) and searched against the Uniprot mouse database using Mascot Server version 2.5 with the following parameters: maximum one missed cleavage site, carbamidomethylation at Cys residues as fixed modification and Met oxidation, Asn, Gln deamidation, and protein *N*-acetylation as variable modifications. Precursor ion mass tolerance was set to 20 ppm, and fragment mass tolerance was set to 0.25 Da.

### Statistical analysis

For quantification of rod outer segment content of Gα_t_ and Gβ_1_ subunits and comparative analysis of retinal morphology, data are presented as the mean ± SD. All statistical analyses were performed with GraphPad Prism 7.04. Morphologic data were analyzed using the nonparametric Mann–Whitney *U* test, and results were considered statistically significant at *p* < 0.05. For the MS identification of G-protein γ-subunits present in rod outer segments, Mascot data were imported into Scaffold 4.8 (Proteome Software) to merge all of the data for a sample represented by multiple gel bands, to estimate a confidence score for protein identification, and to perform a relative protein quantification based on the sum of intensities of the constituent peptides.

## Results

Hereafter, we will refer to the two *Gγ_1_^−/−^* mouse strains analyzed in this study as “Deltagen” and “StL” *Gγ_1_^−/−^* mice, as the mouse described in the study by Lobanova et al. (2008) was produced by and licensed from Deltagen and the mouse characterized in the study by Kolesnikov et al. (2011) was produced at Saint Louis University.

### Identification of transducin α- and β-subunit levels in *Gγ_1_^−/−^* retinal lysates

**Figure 1.**
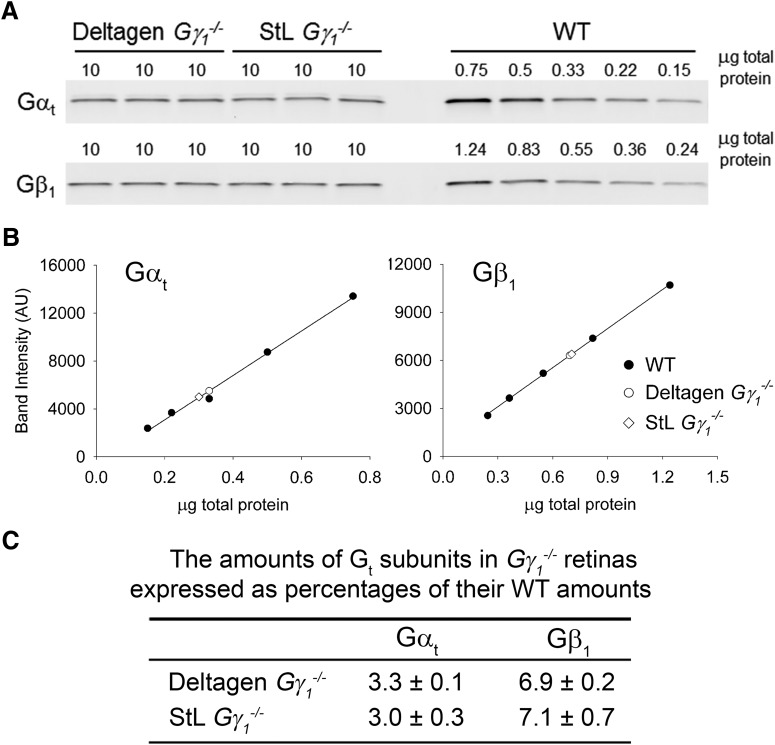
Comparative analysis of Gα_t_ and Gβ_1_ expression levels in retinal lysates from 1-month-old *Gγ_1_^−/−^* mice. ***A***, Retinal lysates from Deltagen and StL mouse strains containing 10 μg of total protein were separated by SDS-PAGE alongside serial dilutions of WT retinal lysates and were immunoblotted with antibodies against each transducin subunit. Data are taken from one of three similar experiments. ***B***, The relative contents of Gα_t_ and Gβ_1_ in retinal lysates analyzed in ***A*** were determined from the calibration curves obtained from the WT lysate dilutions (Gα_t_ regression equation: *y* = 18,600 × *x* − 623; Gβ_1_ regression equation: *y* = 8130 × *x* + 665). • = WT, ○ = Deltagen *Gγ_1_^−/−^*, and ♢ = StL *Gγ_1_^−/−^*. ***C***, The amounts of transducin subunits in *Gγ_1_^−/−^* retinas averaged across three independent experiments are expressed as percentages of their amounts in WT retinas (mean ± SD). The amount of Gβ_1_ was corrected to reflect that only 18% of Gβ_1_ in *Gγ_1_^−/−^* retinas is expressed in rods, whereas the rest is expressed in the inner retina (Lobanova et al., 2008).

We used quantitative Western blotting to directly compare the levels of Gα_t_ and Gβ_1_ in the two Gγ_1_ knock-out models. Retinas were harvested at 1 month of age and lysed, and proteins from the lysates were separated by SDS-PAGE alongside serial dilutions of retinal lysates from WT mice (Fig. 1*A*). Gα_t_ and Gβ_1_ were then detected by immunoblotting with specific antibodies against each protein. The standard curves relating band intensity to total protein amount were obtained from WT lysates and used to calculate relative contents of Gα_t_ and Gβ_1_ in each model (Fig. 1*B*).

This analysis revealed an identical reduction in the contents of transducin subunits in the retinas of both mouse strains (Fig. 1*C*). Gα_t_ was present at 3.3% and 3.0% of the WT amount in Deltagen and StL retinas, respectively, whereas Gβ_1_ was present at 6.9% and 7.1% of the WT amount.^a^ These numbers are very close to those previously reported for the Deltagen mouse (3.9% remaining Gα_t_ and 10.6% Gβ_1_; Lobanova et al., 2008), a conclusion reinforced by the fact that the standard curves in the study by Lobanova et al. (2008) were generated based on an alternative methodology using purified protein standards rather than serial dilutions of WT retinal extracts. It is worth noting that the difference between the conclusions of the two studies could not be attributed to different states of light-dependent transducin translocation, a phenomenon taking place in WT rods (Sokolov et al., 2002), because transducin does not translocate in *Gγ_1_^−/−^* rods (Lobanova et al., 2008). These data clearly demonstrate that the amount of transducin remaining in retinas of the StL mouse was overestimated and that this aspect of the *Gγ_1_^−/−^* phenotype is strain independent.

We next calculated how much Gα_t_ and Gβ_1_ is present in rod outer segments of *Gγ_1_^−/−^* mice, taking into account that approximately one-half of each subunit is mislocalized from *Gγ_1_^−/−^* rod outer segments and a large (∼82%) fraction of Gβ_1_ in this mouse is expressed in the inner retina (Lobanova et al., 2008; Table 1). Therefore, rod outer segments of Deltagen *Gγ_1_^−/−^* mice contain between ∼1.5% (based on the lower value of 3.0% measured in Fig. 1) and ∼2% (based on the higher 3.9% value measured in the study by Lobanova et al., 2008) of WT Gα_t_. The range for the Gβ_1_ amount in *Gγ_1_^−/−^* outer segments is between 0.6% and 1% of its WT content (based on the total retinal amounts of 7% and 10.6%, respectively, obtained in the current and previous study). The corresponding amounts for the StL mice derived from the values reported in Fig. 1 are 1.5% and 0.7% of WT, respectively, for Gα_t_ and Gβ_1_ (Table 1).

**Table 1.** The amounts of transducin subunits in *Gγ_1_^−/−^* **rod outer segments and the corresponding sensitivities of rod light responses expressed as percentages of WT**

	Gα_t_	Gβ_1_	Light sensitivity
Deltagen *Gγ_*1*_^*−/−*^*	1.5-2.0%	0.6-1.0%	1.5%
StL *Gγ_*1*_^*−/−*^*	1.5%	0.7%	1.1%

The mean values for protein content in retinas were taken from Fig. 1 and the study by Lobanova et al. (2008) and were corrected for the outer segment fraction of each protein, as described in the text. Relative light sensitivities were derived from the single-cell recordings in the studies by Lobanova et al. (2008) and Kolesnikov et al. (2011) by dividing the value of half-saturating light intensity of WT rods by the corresponding value for *Gγ_1_^−/−^* rods.

### Consideration of transducin activation mechanism in *Gγ_1_^−/−^* rods

Precise determination of the degree of transducin subunit loss in the outer segments of *Gγ_1_^−/−^* rods allows critical evaluation of the two hypotheses explaining light signaling in these cells: Gα_t_ activated alone versus Gα_t_ activation assisted by an alternative Gβγ. As mentioned above, the reduction in photoresponse sensitivity documented for these cells was consistent between both studies (∼67-fold in Lobanova et al., 2008; ∼90-fold in Kolesnikov et al., 2011). This represents 1.1–1.5% of WT sensitivity and is very close to the degree of Gα_t_ and Gβ_1_ reduction determined in the previous section (Table 1). Because photoreceptor light sensitivity is directly proportional to the concentration of transducin heterotrimer on the membranes of outer segment discs (Pugh and Lamb, 1993; Heck and Hofmann, 2001; Sokolov et al., 2002; Arshavsky and Burns, 2014), the comparable reduction in Gα_t_ and photoresponse sensitivity indicates that the Gα_t_ remaining in *Gγ_1_^−/−^* rod outer segments is activated by rhodopsin at nearly the same efficiency as in WT outer segments. On the other hand, the efficiency of Gα_t_ activation by rhodopsin without Gβγ is at least an order of magnitude lower than with Gβγ (Fung, 1983; Kisselev et al., 1999; Herrmann et al., 2006). This argues that Gα_t_ activation in *Gγ_1_^−/−^* rods is supported by a complex between Gβ_1_ and a G-protein γ-subunit replacing Gγ_1_ in these rods. We therefore set up a search for this alternative Gβ_1_γ complex in *Gγ_1_^−/−^* rods.

### Identification of alternative Gβγ complexes in *Gγ_1_^−/−^* and WT rod outer segments

To elucidate the molecular composition of the putative Gβ_1_γ complexes supporting visual function in the absence of Gγ_1_, we first conducted MS identification of all G-protein γ-subunits present in rod outer segments of WT and *Gγ_1_^−/−^* mice. Outer segments were purified from each mouse type, and their proteins were separated by SDS-PAGE. Gel fragments containing G-protein γ-subunits were excised, and proteins were subjected to in-gel tryptic digestion followed by liquid chromatography-MS/MS analysis of the resulting peptides. Of the 14 G-protein γ-subunits encoded in the mouse genome (Milligan and Kostenis, 2006), only 3 were found in WT and 2 were found in *Gγ_1_^−/−^* outer segments (Table 2). As expected, WT but not *Gγ_1_^−/−^* outer segments contained Gγ_1_. The other two subunits, Gγ_2_ and Gγ_3_, were found in both preparations, although the confidence score for Gγ_3_ in the WT preparation was low.^b^ A rough estimate, based on comparing the total ion intensity produced by all peptides representing each Gγ subunit type, suggested that for every 82 molecules of Gγ_1_ present in WT outer segments there are 3 molecules of Gγ_2_ and 1 molecule of Gγ_3_. A similar estimate performed for *Gγ_1_^−/−^* rod outer segments suggested a molar ratio of ∼1.7 between Gγ_2_ and Gγ_3_. These results narrowed the list of potential Gβ_1_ binding partners in *Gγ_1_^−/−^* outer segments to these two γ-subunits.

**Table 2.** Mass spectrometry identification of G-protein γ-subunits present in rod outer segments of WT and *Gγ_1_^−/−^* **mice**

	Peptides (*n*)	Protein score	Confidence score
WT			
Gγ_1_	6	187	100
Gγ_2_	1	26	99.2
Gγ_3_	2	23	31
*Gγ_*1*_^*−/−*^*			
Gγ_2_	2	25	99.9
Gγ_3_	1	55	99.4

**Table:** Statistical Table

Line	Figure/table	Data distribution	Type of test	*p* value
a	Fig. 1C All data	*N* too small to determine if normally distributed	N/A	N/A
b	Table 2 All data	*N* too small to determine if normally distributed	Confidence score from Scaffold 4.8	N/A
c	Fig. 3B Deltagen vs StL at 1 month	*N* too small to determine if normally distributed	Mann–Whitney *U* test	0.065
d	Fig. 3B Deltagen vs StL at 3 months	*N* too small to determine if normally distributed	Mann–Whitney *U* test	0.041
e	Fig. 3B Deltagen vs StL at 6 months	*N* too small to determine if normally distributed	Mann–Whitney *U* test	0.24

**Figure 2.**
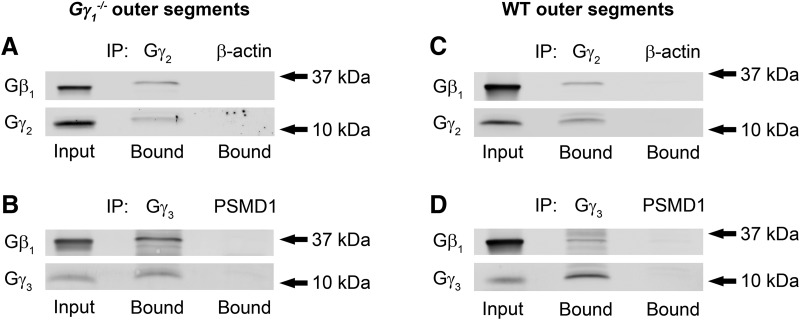
Identification of alternative Gβ_1_γ complexes in rod outer segments of *Gγ_1_^−/−^* and WT mice. ***A–D***, Coimmunoprecipitation experiments were performed by incubating *Gγ_1_^−/−^* (***A***, ***B***) or WT (***C***, ***D***) rod outer segment lysates with mouse anti-Gγ_2_ (***A***, ***C***) or rabbit anti-Gγ_3_ (***B***, ***D***) antibodies. Immunoprecipitation with species-matched anti-β-actin (sc-47778) and anti-PSMD1 (ab140682) antibodies were used as negative controls; these antibodies were chosen based on the lack of cross-reactivity with the proteins analyzed in this panel, as evaluated in independent experiments. The data represent one of four similar experiments performed with *Gγ_1_^−/−^* or two similar experiments performed with WT outer segment preparations.

Therefore, we sought to directly demonstrate that Gβ_1_ forms complexes with Gγ_2_ and/or Gγ_3_ in *Gγ_1_^−/−^* rod outer segments by coimmunoprecipitating these putative complexes using antibodies against Gγ_2_ and Gγ_3_. We have found that a significant fraction of Gβ_1_ in *Gγ_1_^−/−^* rod outer segments was precipitated with antibodies specifically recognizing Gγ_2_ or Gγ_3_ (Fig. 2*A*,*B*). These results confirm that Gβ_1_ remaining in the rod outer segments of *Gγ_1_^−/−^* mice indeed forms complexes with both of these γ-subunits. We also attempted to conduct a reciprocal coprecipitation experiment, but unfortunately were not able to identify a precipitating antibody against Gβ_1_.

We next sought to determine whether these alternative Gβγ complexes only form in the absence of Gγ_1_ or whether they are also present in WT rods. To test this, we repeated coimmunoprecipitation experiments using rod outer segments from WT mice (Fig. 2*C*,*D*). This analysis revealed that, even in the presence of abundant amounts of its cognate partner Gγ_1_, a small portion of Gβ_1_ binds Gγ_2_ or Gγ_3_. This result suggests that Gβ_1_ normally forms complexes with these two alternative γ-subunits in addition to its canonical Gγ_1_ binding partner, although these complexes are not likely to be functionally significant due to their low abundance.

Together, our experiments demonstrate that Gγ_2_ and Gγ_3_ are naturally present in complexes with Gβ_1_ in the WT rod outer segment. When Gγ_1_ is knocked out, these alternative Gβ_1_γ_2_ and Gβ_1_γ_3_ complexes can support light signaling in mutant photoreceptors.

### Evaluation of photoreceptor degeneration in two strains of *Gγ_1_^−/−^* mice

Another discrepancy reported between the Deltagen and StL *Gγ_1_^−/−^* mouse strains was that photoreceptor degeneration in the former progressed significantly faster than in the latter. We therefore performed a quantitative side-by-side comparison of the rate of retinal degeneration in these animals. This was accomplished by counting the number of photoreceptor nuclei in representative segments of the ONL at 1, 3, and 6 months of age. Although this method is more labor intensive than commonly used alternatives (e.g., measuring the ONL thickness or counting the number of nuclei per ONL stack), it provides the most reliable information on the actual number of photoreceptors remaining “alive” in a degenerating retina. This is because nuclear stacks in degenerating retinas could become distorted and hard to quantify, whereas the ONL thickness may change nonproportionally to the actual cell loss.

**Figure 3.**
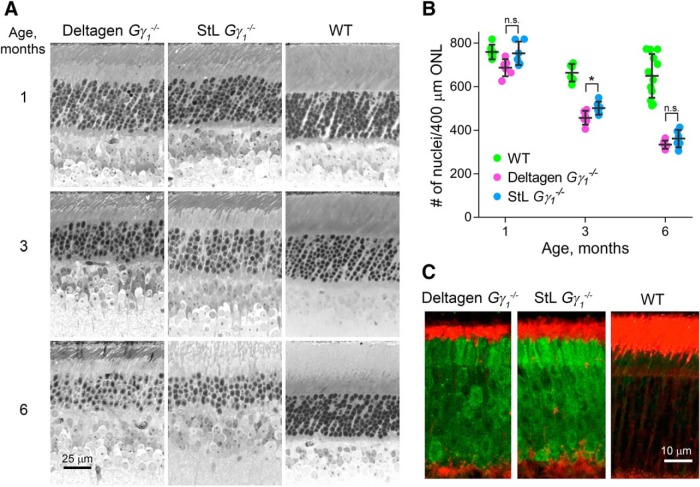
Comparative analysis of retinal morphology in Deltagen and StL *Gγ_1_^−/−^* mice. ***A***, Representative images of toluidine blue-stained, plastic-embedded 1-μm-thick retinal cross sections from each mouse strain at the indicated ages. Scale bar, 25 μm. ***B***, The number of photoreceptor nuclei counted in 400 μm outer nuclear layer (ONL) segments located 1 mm from either side of the optic nerve. The number of nuclei in each segment was counted by hand. Data were averaged across nuclear counts obtained from each side of the optic nerve from three or four animals of each age and genotype, and are shown as the mean ± SD. Each dot represents a single data point. ***C***, Accumulation of the Ub^G76V^–GFP reporter of proteasomal activity (green) in rods of 3-month-old Deltagen and StL *Gγ_1_^−/−^* mice. A Ub^G76V^–GFP-expressing WT retina is shown as a control. Wheat germ agglutinin staining is shown in red. Scale bar, 10 μm.

Retinas from both *Gγ_1_^−/−^* mouse lines were embedded in plastic, cross-sectioned, and stained with toluidine blue (Fig. 3*A*). Photoreceptor nuclei were then counted in two 400 μm segments per eye, one on each side of the optic nerve, and the values were averaged across at least three mice of each age (Fig. 3*B*). These data indicated that the rates of photoreceptor degeneration in both knock-out strains were nearly identical at all tested ages. Formal analysis suggested that a statistically significant difference in nuclear counts existed in 3-month-old mice (*p* = 0.065^c^ at 1 month; *p* = 0.041^d^ at 3 months; *p* = 0.24^e^ at 6 months). However, the absolute difference of <10% is hard to consider physiologically significant. This analysis showed that approximately half of the photoreceptor cells were lost by the age of 6 months, a rate of degeneration that falls somewhere in the middle between the two previous reports. One potential source of this discrepancy is that the first study (Lobanova et al., 2008) did not assess this parameter quantitatively and apparently provided more dramatic examples of cellular loss than average. On the other hand, the rate of photoreceptor degeneration in the study by Kolesnikov et al. (2011) may have been underestimated due to the use of the nuclear stack counting methodology, which may be more arbitrary than the total nuclear count.

### Photoreceptors from both strains of *Gγ_1_^−/−^* mice experience proteostatic stress

Using the Deltagen strain, Lobanova et al. (2013) showed that photoreceptor cell death in *Gγ_1_^−/−^* mice is associated with proteostatic stress arising from the necessity to degrade large amounts of Gβ_1_ that is unable to fold without its constitutive Gγ_1_ partner. We now demonstrate that the same is true for the StL strain. StL mice were crossed with the mouse ubiquitously expressing the Ub^G76V^–GFP proteasome activity reporter, which consists of GFP fused to an uncleavable ubiquitin (Lindsten et al., 2003). This reporter undergoes efficient polyubiquitination and proteasomal degradation in healthy cells, including WT photoreceptors, but accumulates in cells suffering from impairment or insufficiency of the ubiquitin–proteasome system. Consistent with the phenotype of the Deltagen strain, photoreceptors of StL mice also displayed robust intracellular accumulation of Ub^G76V^–GFP, as documented by detecting GFP fluorescence in retinal cross sections (Fig. 3*C*). This result indicates that both mouse strains suffer from abnormal proteostasis, contributing to their photoreceptor degeneration.

## Discussion

The data presented in this study demonstrate that the two lines of Gγ_1_ knock-out mice currently available to the scientific community display essentially identical phenotypes. Not only do they produce similar responses to light, as reported earlier, but they also contain identical amounts of Gα_t_ and Gβ_1_ subunits in their rods, undergo retinal degeneration at a similar rate, and share the same underlying pathobiological mechanism. Therefore, these strains could be used interchangeably in future studies of this mouse model.

Most importantly, resolving the discrepancy between previous estimates of the contents of transducin subunits in these strains allowed us to explain the mechanism by which photoreceptors respond to light in the absence of the transducin canonical βγ-subunit Gβ_1_γ_1_. Here we demonstrate that relatively small amounts of Gγ_2_ and Gγ_3_ are endogenously expressed in the outer segments of these cells where they produce complexes with Gβ_1_. These complexes are able to support vision in the absence of the Gβ_1_ cognate γ-subunit Gγ_1_.

Our findings highlight the versatility of G-protein signaling by showing the exchangeability of individual G-protein subunits in performing an important physiologic function. Mouse and human genomes contain genes encoding 5 G-protein β-subunits and 14 γ-subunits (Milligan and Kostenis, 2006), and their expression patterns in different tissues vary widely (Largent et al., 1988; Gautam et al., 1990; Cali et al., 1992; Liang et al., 1998; Morishita et al., 1998). Given that many possible Gβγ combinations exist, a key question in G-protein signaling is whether heterotrimers composed of distinct subunits can fulfill the same physiologic role. Many examples documented in cell culture and *in vitro* show that Gβγ complexes using different γ-subunits are able to activate the same signaling cascades (Wickman et al., 1994; Ikeda, 1996; Ruiz-Velaso and Ikeda, 2000). This includes at least three cell culture studies specifically demonstrating the functional interchangeability of Gβ_1_γ_1_, Gβ_1_γ_2_, and Gβ_1_γ_3_ (Hawes et al., 1995; Dingus et al., 2005; Poon et al., 2009).

Examination of γ-subunit exchangeability in the context of the whole animal has thus far been limited, but results obtained in several mouse knock-out studies have suggested that there may be a more stringent requirement for γ-subunit specificity *in vivo* than in cell culture. For example, Gγ_7_ is indispensable for adenylyl cyclase signaling through the A_2A_ receptor in the striatum (Schwindinger et al., 2003, 2010), whereas Gγ_13_ is required for olfactory signal transduction (Li et al., 2013).

We now provide a compelling example of the functional interchangeability of G-protein γ-subunits *in vivo*. To our knowledge, *Gγ_1_^−/−^* represents the first case to directly demonstrate that multiple Gβγ complexes can perform the same function in a living animal. The only caveat is that the expression levels of alternative γ-subunits in photoreceptors are lower than those of the conventional Gγ_1_ and, therefore, the Gβ_1_γ_1_ complex drives the majority of phototransduction unless Gγ_1_ is absent.

## Synthesis

Reviewing Editor: Siegrid Löwel, University of Goettingen Decisions are customarily a result of the Reviewing Editor and the peer reviewers coming together and discussing their recommendations until a consensus is reached. When revisions are invited, a fact-based synthesis statement explaining their decision and outlining what is needed to prepare a revision will be listed below. The following reviewer(s) agreed to reveal their identity: Karin Dedek, Peter Calvert

The manuscript #eN-NWR-0144-18 describes careful experiments that resolve an important controversy in the field of vertebrate phototransduction, and in G protein coupled receptor signaling in general. In particular, the study resolves a controversy on the interpretation of results obtained from Gγ1 knockout mouse in vitro and show that canonical (β1γ1) and non-canonical combinations of β1 and γ2,3 subunits can fulfill the same functions in vivo.

The heterotrimeric G protein transducin is important for phototransduction in vertebrate photoreceptors. Two different mouse lines exist in which the gamma1 subunit has been knocked out. Both studies showed strong reduction in light sensitivity but came to different conclusions on the pathomechanism. One study proposed that the remaining Gαt subunit is sufficient for phototransduction while the other study claimed that Gαt binds to remaining Gβ1 and non-canonical γ2 or γ3 subunits. The authors of the present study set out to resolve this issue and compared both mouse strains side-by-side.

Using Western blot experiments, MALDI analysis and co-immunoprecipitation they reveal that both mouse strains show very similar phenotypes, express similar residual amounts of Gαt and Gβ1 subunits, and contain complexes of Gβ1 and non-canonical γ subunits. Additionally, they studied the time course of retinal degeneration and found - in contrast to earlier studies - a similar time course and pathomechanism in both mouse strains.

The study is well designed and performed. The quality of the data is spectacular, the results are presented in a clear and cogent way and the discussion is very fair - equally identifying shortcomings in the original works of both groups as leading to the disparate conclusions. The manuscript thus represents an important step in regularizing the conclusions of the two groups and establishes an important president for interchangeability of G protein beta/gamma subunits within a functioning neuron. Additionally, their results suggest that canonical (β1γ1) and non-canonical combinations of β1 and γ2,3 subunits can fulfill the same functions in vivo.

There are only some minor comments and suggestions the authors should answer/comment on when resubmitting their manuscript:

1. The results from co-immunoprecipitation and Western blot analyses strongly rely on the subunit specificity of the antibodies used. The authors should comment on this. Please also give a rationale for why PSMD1 and actin are good negative controls for co-IP experiments.

2. Methods: Please give the composition of the mouse Ringer's solution.

3. Number of photoreceptor nuclei: Most likely the authors counted photoreceptor nuclei in a retinal area. They should give the area size in µm^2.

4. Transducin undergoes light-dependent translocation in rods. Could differences in experimental layout (time of day etc) also account for the differences in the two earlier studies (Lobanova et al., 2008; Kolesnikov et al., 2011)? The authors should comment on this.

## Author Response

1. The results from co-immunoprecipitation and Western blot analyses strongly rely on the subunit specificity of the antibodies used. The authors should comment on this.

A: The following statement is added to the Methods section: "The specificity of the anti- Gαt antibody in the context of retinal tissue was directly tested in Gαt knockout animals. The specificity of other antibodies was assumed per manufacturer's descriptions."

Please also give a rationale for why PSMD1 and actin are good negative controls for co-IP experiments.

A: These are not actin and PSMD1, but rather species-matched antibodies against these proteins. As far as these control antibodies do not specifically precipitate the proteins of interest (G protein subunits in our case) the nature of their antigen recognition is not relevant to this analysis. These are controls for non-specific protein binding by an antibody raised in the same species as experimental antibodies. Since all anti-Gβ1, -Gγ2 and -Gγ3 AB were commercial and, therefore, pre-immune IgG were not available as controls, we used high quality antibodies available in our lab raised in the corresponding species (which happened to be mouse AB against actin and rabbit AB against PSMD1). Both control AB were tested not to cross react with proteins of interest. A brief statement of their lack of cross-reactivity is added to the Figure 2 legend.

2. Methods: Please give the composition of the mouse Ringer's solution.

A: The requested information is added to the Methods section.

3. Number of photoreceptor nuclei: Most likely the authors counted photoreceptor nuclei in a retinal area. They should give the area size in Âµm^2.

A: As indicated in the text, we counted the nuclei located specifically in the outer nuclear layer (ONL), not the whole retina. The ONL nuclei represent exclusively photoreceptor cell nuclei. Because photoreceptor degeneration is associated with the loss of these nuclei, the area they occupy is also reduced, as clear from the images in Figure 3a. As we thoroughly discuss in the manuscript, measuring the area occupied by the nuclei of surviving photoreceptors instead of counting their exact number (aka the number of surviving photoreceptor cells) could provide an aberrant quantitative account of degeneration and may vary in different mouse background strains. In fact, the examples shown in Figure 3a illustrate a very modest (if any) decrease in the size of the ONL area in 3-mo-old StL mice compared to WT control despite an ~35% reduction in the nuclear count (Figure 3b). Therefore, we believe that any reference to µm^2 may be potentially misinterpreted and should not be mentioned in this context. This opinion follows the convention in the retinal degeneration field.

4. Transducin undergoes light-dependent translocation in rods. Could differences in experimental layout (time of day etc) also account for the differences in the two earlier studies (Lobanova et al., 2008; Kolesnikov et al., 2011)? The authors should comment on this.

A: The intracellular localization of Gαt and Gβ1 in light- and dark-adapted Gγ1-/- rods was specifically investigated in Lobanova et al. (2008). This analysis showed that both Gαt and Gβ1 are distributed throughout the whole length of both dark- and light-adapted Gγ1-/- rods and neither subunit undergoes light-dependent translocation in the absence of Gγ1. Thus, subunit translocation due to time of day or laboratory light conditions would not have affected the results of either study. A brief statement regarding transducin translocation in WT and Gγ1-/- rods is added to the Results section (lines 202-205).
